# Primary Systemic Al Amyloidosis Presenting as Temporal Arteritis

**DOI:** 10.1155/2014/549641

**Published:** 2014-01-19

**Authors:** Hakan Emmungil, Melike Kalfa, Burcu Başarık, Hasip Kahraman, Ferhat Tanhan, Banu Yaman, Atiye Öztürk, Zehra Erdemir, Gülşen Kandiloğlu, Vedat İnal, Yasemin Kabasakal

**Affiliations:** ^1^Mersin State Hospital, Department of Internal Medicine, Division of Rheumatology, Nusratiye Hometown, Kuvayi Milliye Street No. 32, 33050, Turkey; ^2^Division of Rheumatology, Department of Internal Medicine, Mardin State Hospital, Vali Ozan Street, Mardin, Turkey; ^3^Department of Pulmonology, Ege University Faculty of Medicine, Ege University Hospital, Ankara Street, 35040 Izmir, Turkey; ^4^Department of İnfectious Disease, Ege University Faculty of Medicine, Izmir, Turkey; ^5^Ege University Faculty of Medicine, Ege University Hospital, Ankara Street, 35040 Izmir, Turkey; ^6^Department of Pathology, Ege University Faculty of Medicine, Ege University Hospital, Ankara Street, 35040 Izmir, Turkey; ^7^Division of Rheumatology, Department of Internal Medicine, Ege University Faculty of Medicine Ege University Hospital, Ankara Street, 35040 Izmir, Turkey

## Abstract

Temporal arteritis is most common vasculitis in elderly and imitated by miscellaneous disorders. Temporal artery biopsy is the gold standard test in the diagnosis of giant cell arteritis (GCA). Hereby, we describe a case of a 67-year-old man who presented initially with temporal arteritis; however, a lip biopsy then revealed AL amyloidosis. In this respect, temporal artery biopsy should be performed for definitive diagnosis of GCA particularly patients with systemic symptoms and treatment resistant.

## 1. Primary Systemic Al Amyloidosis Presenting with Temporal Arteritis

Giant cell arteritis (GCA), also called temporal arteritis, is a vasculitis that affects large-and middle-sized blood vessels in individuals older than 50 years of age. A temporal artery biopsy (TAB) is the gold standard test in the diagnosis of GCA [1]. Because corticosteroid therapy is required for more than 1 year in most cases, the pathological confirmation of this vasculitis is advisable. AL amyloidosis is a systemic disorder that can present with a variety of symptoms or signs, including heavy proteinuria, edema, hepatosplenomegaly, otherwise, unexplained heart failure, and the carpal tunnel syndrome [2]. It often requires high clinical suspicion because symptoms are miscellaneous and easily mimicked by more common disorders. Hereby, we describe a case of a 67-year-old man who presented initially with GCA; however, a minor salivary gland biopsy then revealed AL amyloidosis. Hence, temporal artery biopsy should be performed for definitive diagnosis of temporal arteritis.

## 2. Case Report 

**Figure 1:**
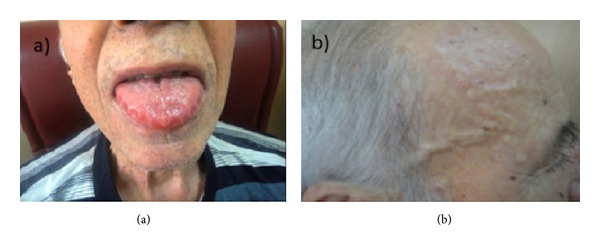
(a) Macroglossia due to amyloidosis and (b) enlarged and nodular temporal artery.

A 67-year-old man was applied to our hospital with a one-year history of weakness, fatigue, headache, extremity, and jaw claudication. Two months earlier he developed bilateral lack of vision and diagnosed ischemic optic neuropathy by ophthalmologist and hence started high dose corticosteroid treatment. Eye findings were improved by this treatment. On examination, the patient appeared well and his vital signs were normal. The temporal arteries were noted to be thickened, nodular, and decreased pulsation (Figure 1). Temporal arteritis was considered because of these findings and methotrexate was added to the patient therapy. Temporal artery biopsy was not considered in consequence of receiving corticosteroid therapy for two months.

During the followup, just after corticosteroid dose reduced, the patient was hospitalized to reevaluate because of recurrent complaints of increasing difficulty in walking, claudication, headache, fatigue, and rising again erythrocyte sedimentation rate (ESR) values. In addition to the preceding findings, the patient presented with macroglossia, hard nodular structures of lips, buccal dryness, livedo reticularis in the back skin and apical systolic murmur. The result from a complete blood cell count was normocytic normochromic anemia. Renal function, albumin, globulin, and calcium levels were all within normal ranges. Ultrasonographic evaluation of the temporal artery and axillary and lower extremity arteries revealed thickened vessel walls. An echocardiogram demonstrated that biatrial dilatation, global ventricular hypokinesia. Cranial MRI taken because of syncope was compatible with subacute infarct.

**Figure 2:**
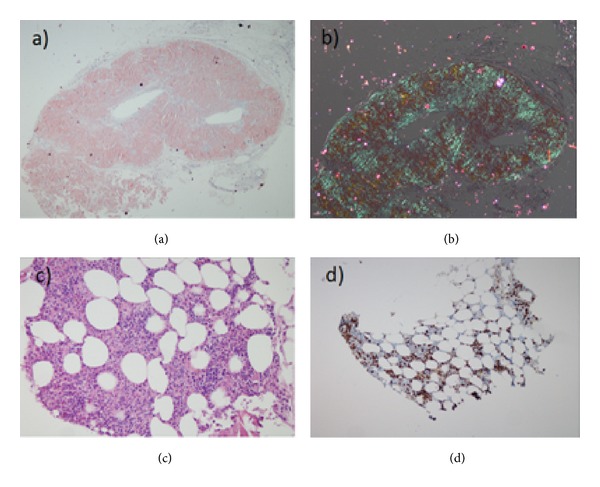
(a) Homogenous accumulation of amyloid fibrilar proteins around the minor salivary gland, (b) polarized light microscopy green amyloid deposition in the lip biopsy, (c) increased atypical plasma cell, and (d) lambda(*λ*) light chain in the bone marrow biopsy.

Vascular deposition disease may be a consideration because of generalize vascular findings. Since the patient had macroglossia and buccal dryness, the lip biopsy was performed from nodular structures. Hematoxylin and eosin staining of the biopsy showed concentric intimal thickening and deposition of large amorphous, eosinophilic material, suggestive of amyloid deposits in the media of the arteries (Figure 2). Histochemical examination was positive for congo red staining and result amyloid angiopathy (Figure 2). Further investigations were performed on the basis of a diagnosis of AL amyloidosis. Electrophoresis did reveal a monoclonal spike (Immunoglobulin G lambda) and free lambda (*λ*) light chains 213 mg/dL (93–242) with a kappa/lambda ratio of 0.24. A bone-marrow aspiration showed 30% atypical plasma cells, which revealed multiple myeloma. Skeletal X-rays revealed that multiple lytic lesions and bilateral femur and humerus bones. In preparing for chemotherapy of myeloma the patient died due to acute myocardial infarction. Patient had myocardial infarction due to a high probability cardiac amyloidosis as a facilitating factor.

## 3. Discussion

Herein, we described a patient who presented with classic jaw claudication, ocular symptoms, headache, and thickened temporal artery mimicking GCA and diagnosed primary systemic amyloidosis.

Primary systemic, or light chain amyloidosis (AL) is characterized by a clonal population of plasma cells in the bone marrow that produce monoclonal light chain of kappa or lambda type. Amyloidogenic light chains misfold forming a highly ordered beta pleated sheet configuration which is the structure that defines amyloid fibrils of any type (including light chain, hereditary, senile systemic or secondary). Contiguous beta pleated sheets wind together into a fibrillar configuration instead of the typical alpha helical pattern of most proteins [3]. Amyloid fibrils deposit in organs, progressively interfering with organ structure and function [4, 5]. Commonly affected organs include the heart, kidneys, gastrointestinal tract/liver, or the peripheral or autonomic nervous system (NS).

AL amyloidosis should be suspected in any patient with a monoclonal gammopathy and unexplained shortness of breath, fatigue, edema, weight loss, orthostasis, or paresthesias [5]. However, it often requires a watchful clinician because symptoms are diverse and easily mimicked by more common disorders.

There have been other case reports documenting the confusion between GCA and AL, when AL presented with atypical manifestations of that mimic GCA. In one report a patient had symptoms suggestive of GCA including nightly and daily scapular girdle and neck pain with morning stiffness. Temporal arteries seemed clinically normal and biopsy of the temporal artery disclosed AL type amyloidosis [6]. Salvarni et al. presented 3 cases of AL amyloidosis in another report that before more typical symptoms of AL were present they developed GCA specific manifestations such as headache, proximal muscle weakness, lower extremity claudication [7]. Churchill et al. reported 2 patients with symptoms of jaw claudication and visual disturbance like our patients suggestive of GCA [8]. Both had anemia and an elevated ESR. The diagnosis that was made by a temporal artery biopsy revealed amyloid deposition in one patient; in the second patient worsening renal function and proteinuria led to a renal biopsy that confirmed amyloid deposition. The temporal artery biopsy was also positive for amyloid in the second patient [8].

In the literature, except for the cases described above AL mimicking GCA, also cases of an association between AL amyloidosis and GCA have been reported [7, 9]. In one case, a temporal biopsy revealed multinucleated giant cells that were engulfing amyloid deposit [9]. The patient presented with weight loss, weakness, temporal and occipital headaches, polyarthralgias, and myalgias. A bone marrow biopsy was diagnostic for multiple myeloma. Two years later she developed jaw claudication, blurred vision, and prominent temporal arteries. A temporal artery biopsy revealed multinucleated giant cells and a diffuse mononuclear cell infiltrate. In another report, a patient with multiple myeloma-associated AL also had granulomatous arteritis in addition to amyloid on temporal artery biopsy. A diagnosis of both diseases was made [7].

Similar laboratory abnormalities can also be seen in both GCA and AL and such as anemia, elevated acute phase reactants. These can further complicate the clinical story, especially in a patient with AL who presents initially with symptoms of GCA. Therefore, serum and urine immunoelectrophoresis tests may guide us to distinguishing these two diseases.

TAB is the cornerstone of diagnosis and often remains positive for two to six weeks after the commencement of treatment. While imaging modalities [10, 11] such as Doppler ultrasound offer a potential noninvasive means of diagnosing the disease, temporal artery biopsies remain the gold standard for diagnosis of GCA. However, due to the segmental inflammatory involvement of the temporal artery, a contralateral biopsy may be required in patients with high clinical suspicion of GCA [1]. In addition to the confusion between the clinical symptoms of GCA and AL amyloidosis, histologic findings in patients with amyloidosis can mimic a giant-cell lesion. Therefore, it is very important to assess congo red staining to avoid misdiagnosis of GCA particularly without any typical biopsy findings [1].

In conclusion, patients with GCA and AL can share similar clinical and laboratory features including elevated ESR, anemia, and histopathologic findings. Moreover, both diseases may also respond to corticosteroid treatment transiently. In this respect, temporal artery biopsy should be performed for definitive diagnosis of temporal arteritis and especially in patients without granulomatous inflammation investigated for AL amyloidosis via amyloid staining.
